# A proof-of-concept study for developing integrated two-photon microscopic and magnetic resonance imaging modality at ultrahigh field of 16.4 tesla

**DOI:** 10.1038/s41598-017-02864-0

**Published:** 2017-06-02

**Authors:** Meng Cui, Yifeng Zhou, Bowen Wei, Xiao-Hong Zhu, Wei Zhu, Mark A. Sanders, Kamil Ugurbil, Wei Chen

**Affiliations:** 1 School of Electrical and Computer Engineering, Department of Biological Sciences, Purdue University, West Lafayette, Indiana United States; 2 Center for Magnetic Resonance Research, Department of Radiology, University of Minnesota, Minneapolis, Minnesota United States; 3 Department of Neuroscience, University of Minnesota, Minneapolis, Minnesota United States

## Abstract

Functional magnetic resonance imaging (fMRI) based on the blood oxygen level dependent (BOLD) contrast has gained a prominent position in neuroscience for imaging neuronal activity and studying effective brain connectivity under working state and functional connectivity at resting state. However, the fundamental questions in regards to fMRI technology: how the BOLD signal inferences the underlying microscopic neuronal activity and physiological changes and what is the ultimate specificity of fMRI for functional mapping of microcircuits, remain unanswered. The capability of simultaneous fMRI measurement and functional microscopic imaging in a live brain thus holds the key to link the microscopic and mesoscopic neural dynamics to the macroscopic brain activity at the central nervous system level. Here we report the first demonstration to integrate high-resolution two-photon fluorescence microscopy (TPM) with a 16.4 tesla MRI system, which proves the concept and feasibility for performing simultaneous high-resolution fMRI and TPM imaging at ultrahigh magnetic field.

## Introduction

Human brain is central to control body physiology and action, consciousness and behavior. Understanding human brain function and dysfunction is the key challenge in neuroscience research, and is also essential for the diagnosis and treatment of brain disorders. However, gaining precise knowledge of neural circuits, neuronal signaling process and functional connectivity within and among various circuits in resting or working brain relies on innovative and transformative neuroimaging tools for quantitative and noninvasive assessment of neuronal process and dynamics in live brain across multiple spatiotemporal scales from microscopic, mesoscopic to macroscopic level.

Functional magnetic resonance imaging (fMRI) based on the blood oxygenation level dependent (BOLD) contrast^1–5^ has gained a prominent position in neuroscience for imaging brain activation and studying effective connectivity during task performance^6^. Moreover, the same fMRI technique can be employed to map the functional connectivity of various neural networks based on the temporal coherence of low-frequency spontaneous BOLD fluctuation^7–13^ in a brain at rest state. It is the only neuroimaging modality that provides adequate spatial resolution and sensitivity reaching the intermediate mesoscopic domain for noninvasively mapping and deciphering the human brain activation down to cortical^14–18^ and subcortical cellular layers^19^ and functional columns^18, 20–26^ that serve as the neural computational units in microcircuits of the brain. However, a fundamental neuroscience question related to the fMRI methodology, i.e., how the BOLD signal inferences the underlying neural activity and the associated neurophysiological changes in space and time, remains unanswered, especially at microscopic and mesoscopic scales. The BOLD contrast originates from the magnetic susceptibility effect induced by the oxygenation level change of the hemoglobin inside blood vessels, thus, having a relatively slow response time to neuronal activity change in a range of few seconds^1–5^, and its quantity is determined by the complex interplays of the hemodynamic changes in cerebral blood flow and volume and the oxygen metabolic rate change driven by either evoked or spontaneous neuronal activity^1, 7, 27, 28^. Although the fMRI BOLD signal has served as a surrogate of the brain activity, its quantitative transformation to the underlying neural activity can vary significantly at different scales and brain conditions. Substantial research efforts have been dedicated to study the neural and cellular mechanisms underlying the fMRI BOLD signal.

A common approach used in the literature is to collect electrophysiological signals using implanted metal electrode(s) during the fMRI acquisition, thus, providing simultaneous measurements of fMRI BOLD signals with three types of neuronal activities, *i*.*e*., the single or multiple unit activity and local field potential^27, 29–32^. However, this invasive approach induces a large susceptibility difference between the electrode and brain tissue and severe fMRI signal cancelation in the brain tissue surrounding the electrode. The affected brain region is far beyond the spatial scale and size of neural computational units; thus, makes it impossible to investigate the electrophysiology-BOLD correlation in the same population of neurons at microcircuit level. In addition, the current state-of-the-art neuro-recording method is limited to only a small number of neurons and could potentially lead to biased conclusions when correlating with the fMRI signals, given the fact of more than 20,000 neurons^33–35^ and a plethora of neurocircuit components within a single high-resolution fMRI voxel of 1 mm^3^ in the human cortex.

One alternative and powerful imaging approach for studying neuronal and cellular activities *in vivo* is two-photon fluorescence microscopy (TPM), which has been widely adopted in neuroscience research^36–48^. The excellent spatiotemporal resolutions and the wide selection of functional indicators make TPM well suited for delineating the function and principle of neural network covering a much larger neuron population than the traditional electrophysiology recording methods^41, 49–55^. In addition, the spatial resolution of the TPM is several orders of magnitude higher than that of fMRI. The state-of-the-art TPM can routinely achieve a resolution of ~300 nm, which can clearly resolve the dendritic spines, neurons, astroglial cells and microvessels *in vivo* ^37^. Therefore, the capability of performing simultaneous TPM and fMRI measurement can not only bridge the imaging modalities, but also provide a comprehensive view of the brain with TPM at microscopic and/or mesoscopic scale for understanding the electrophysiology origin of the fMRI signal at mesoscopic and/or macroscopic scale. To develop such a powerful multimodal neuroimaging system for broad neuroscience applications, however, several technical challenges need to be considered and addressed.

First, MRI operating in high magnetic field environment prohibits any magnetic materials and many delicate electronic devices or actuators used in the conventional TPM systems. Second, the MRI acquisition with many on-and-off gradients and radiofrequency (RF) pulses must not interfere the TPM operation and data collection. Third, the setup of the TPM optical device near the brain of interest is constrained by the limited space inside a small-bore animal magnet, and the TPM excitation and emission lights must not be blocked by the RF coil or other components used inside the MRI magnet. For these reasons, conventional TPM systems are incompatible and incapable to work with MRI. Despite the early attempt of combining optical measurement with MRI^56^, there is still no report of TPM imaging with micron or sub-micron level resolution inside an MRI scanner to date. In this work, we aimed to address these technical challenges and conducted the proof of concept study for developing the first MRI-compatible, integrated TPM-MRI multimodal imaging system at ultrahigh magnetic field strength of 16.4 tesla (T) at micron level of TPM spatial resolution.

## Results

### Design concept of the MRI-compatible TPM system at ultrahigh magnetic field

**Figure 1:**
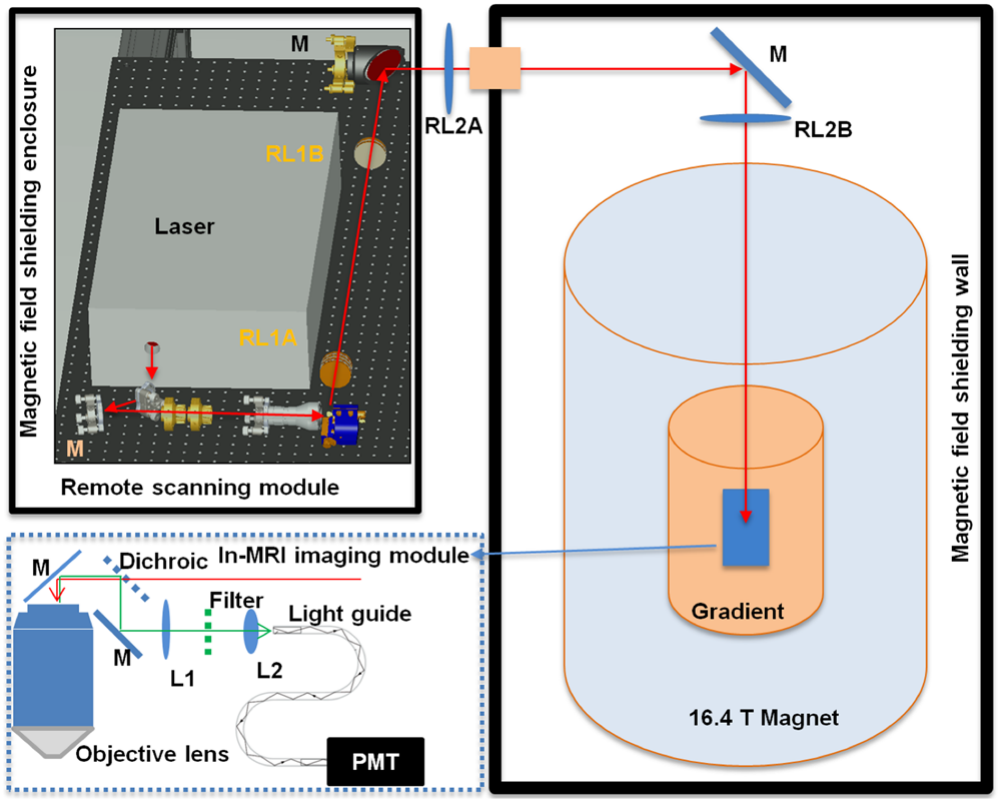
Schematic of the MRI-compatible TPM imaging system. The laser scanning module (upper left corner) was located outside the 16.4T magnet room and contained inside a magnetic field shielding enclosure. The excitation laser beam was guided to the in-MRI imaging module inside the magnet by two sets of relay lenses (RL1A, RL1B, RL2A and RL2B) and mirrors (M). The in-MRI imaging module (enclosed by the blue dotted box) comprised an objective lens, a dichroic beam splitter, an optical mirror, a filter and two lenses (L1 and L2) which focused the two-photon excited fluorescence emission onto the entrance port of a 9 meter long fiber light guide. The exit port of the light guide was in contact with the front window of a GaAsP PMT housed inside the enclosure of the remote scanning module.

We have designed a long-distance remote TPM-MRI multimodal imaging system that can function properly in very high magnetic field environment. The novel TPM system comprised a remote laser scanning system and an in-MRI optical imaging module. The remote laser scanning system was located in a room adjacent to the MRI magnet room with the magnetic field shielding wall (Fig. 1). To ensure the proper functioning of many delicate electronics, actuators, and the laser system, we placed these components on a table supported by a customized frame and covered by a magnetic field shielding enclosure. Beside the laser, the remote laser scanning system comprised three key units: beam control, Galvo scanner, and relay lenses. The beam control unit allowed the adjustment of the laser beam intensity and expanded the laser beam to overfill the Galvo scanning mirror. The Galvo scanner achieved the function of scanning the laser beam in the transversal directions. As the working principle of Galvo relies on the magnetic force, the surrounding magnetic field was reduced to the earth’s field level by the shielding enclosure. The relay lenses included two pairs of telecentric scan lenses. The first pair of lenses was of different focal length, which magnified the laser beam to overfill the objective lens’s pupil. The second pair of lenses had large clear aperture (~150 mm) and identical long focal length (~2 meters) to relay the output of the first lens pair to the back focal plane of the objective lens.

The in-MRI TPM imaging module (dotted box in Fig. 1) consisted of an objective lens, a dichroic beam splitter, an optical mirror, a bandpass optical filter for green fluorescent protein (GFP), two lenses, and the entrance port of a 9-meter long light guide. The objective lens contained only glass, aluminum and brass and thus showed no attraction by the ultrahigh 16.4T (164,000 gauss) magnetic field. As the photomultiplier (PMT) detector routinely used to collect the two-photon excited fluorescence emission is incompatible with the 16.4T magnetic field, we employed the two lenses of the in-MRI module to image the pupil of the objective lens onto the entrance port of a 9-meter long light guide, which delivered the light back to the PMT housed inside the magnetic field shielding enclosure located in the adjacent room. The entire in-MRI module only contained glass, brass, plastic and aluminum, fully compatible with the 16.4T MRI system.

### Imaging performance

**Figure 2:**
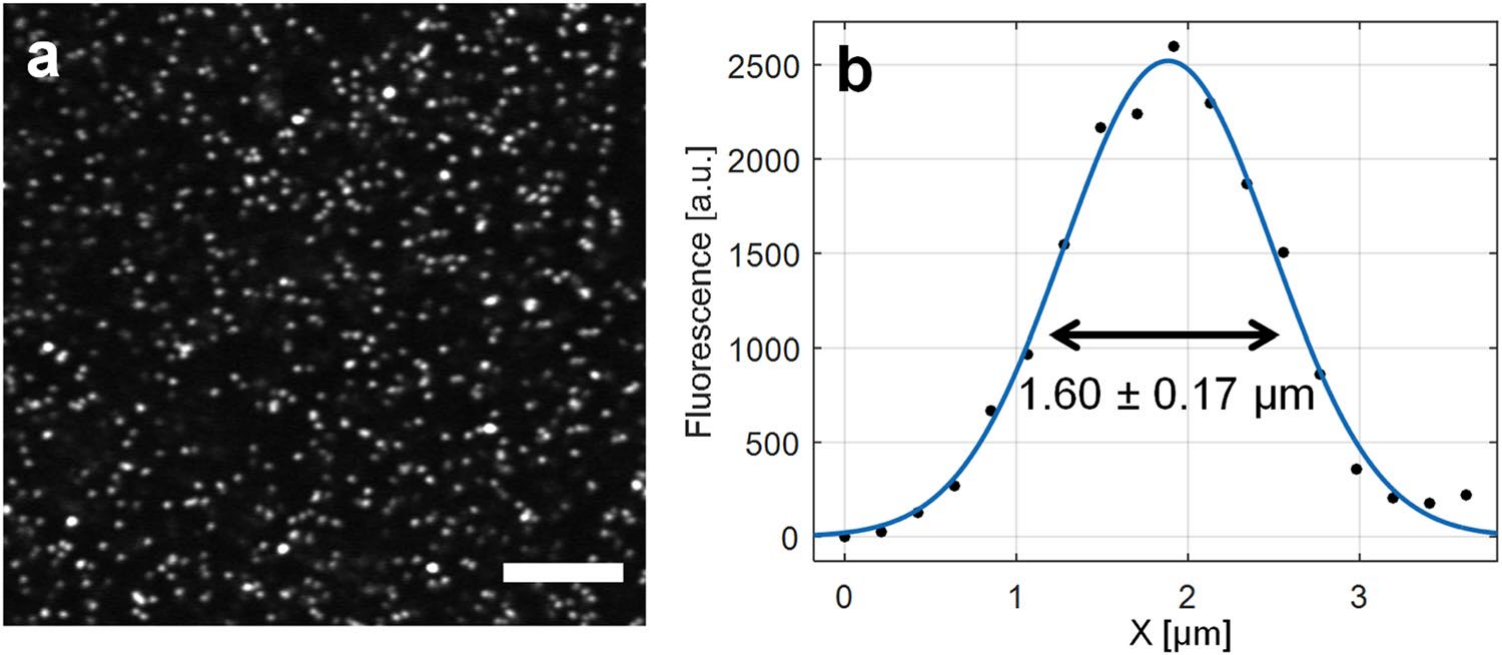
Resolution characterization of the long-distance remote TPM imaging system. (**a**) Two-photon fluorescence images of beads of 1 μm in diameter. Scale bar: 50 μm. (**b**) Plot across the diameter of a bead and its Gaussian fitting (blue line) to determine the FWHM values.

As the overall optical path of the long-distance remote TPM system was very long (~10 meters) with two pairs of relay lenses and the imaging system was not supported by a floated optical table, we need to evaluate whether the overall TPM-MRI system can deliver high imaging quality. To test the imaging performance, we used fluorescence beads of 1 micron (μm) in diameter as the standard sample to quantify the TPM imaging spatial resolution. In theory, the employed objective lens (10X NA 0.26 Mitutoyo Plan Apo NIR) can achieve 1.35 μm in two-photon excitation full-width-at-half-maximum (FWHM) at 930 nm wavelength. Experimentally, we observed 1.60 ± 0.17 μm (n = 10) in FWHM (Fig. 2) when measuring the beads. Considering the finite size of the 1 μm diameter beads, the achieved resolution was near the diffraction-limited value, which indicates that the long-distance remote TPM-MRI imaging system can achieve high-quality imaging with minimal aberration or distortion.

**Figure 3:**
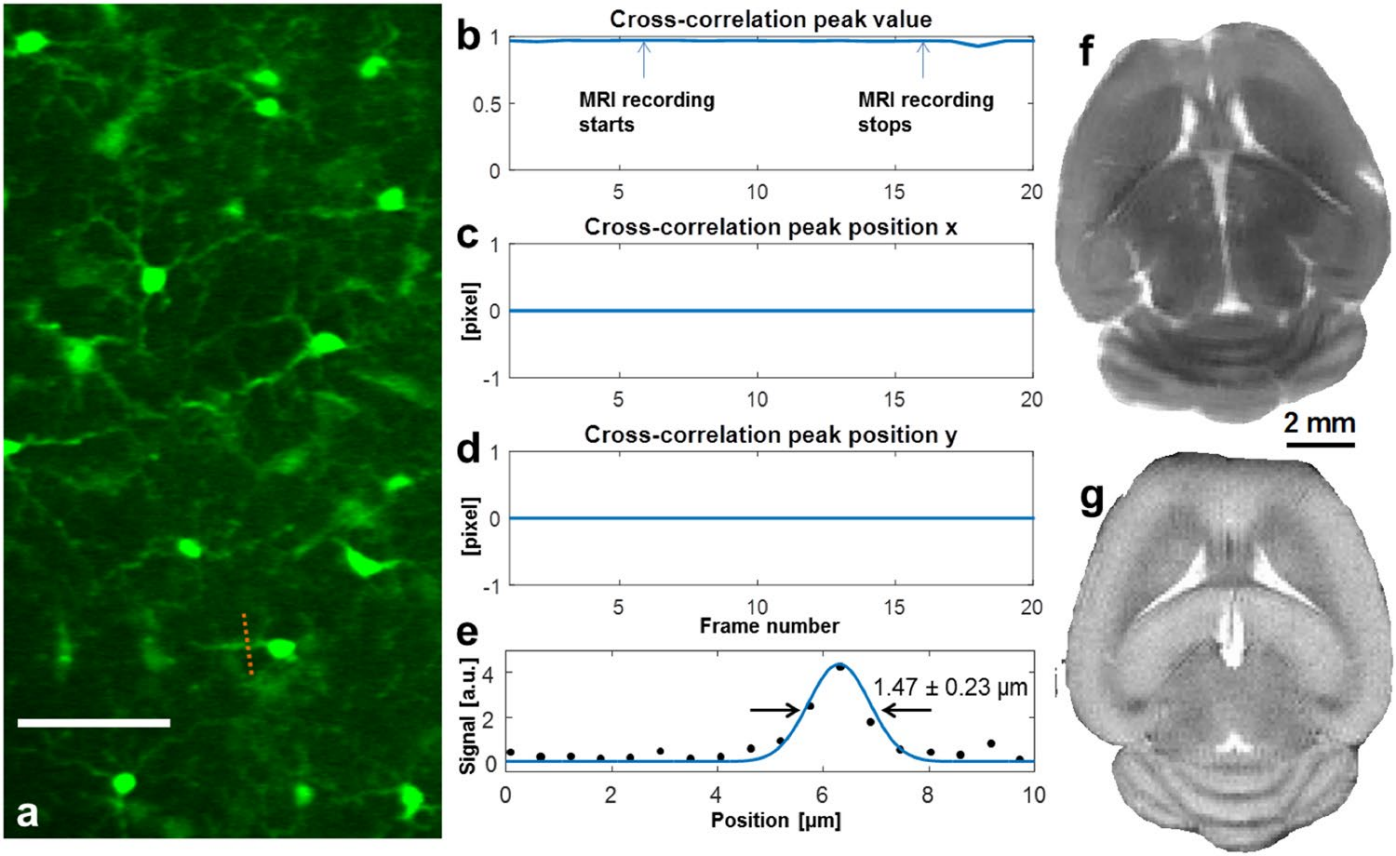
TPM imaging inside the 16.4T MRI magnet. (**a**) TPM images of microglia cells in a fixed CX3CR1 mouse brain (scale bar: 50 μm). (**b**–**d**) The cross-correlation peak value and peak positions of the time lapsed TPM images during which we performed MRI recording. The cross-correlation shows that the operation of MRI had no influence on TPM. (**e**) Cross-sectional plot of the process of microglia and its Gaussian fitting (blue line) to determine the FWHM values. (**f**) *Ex vivo* ultrahigh-resolution spin-echo multiple-slice MRI of the same fixed mouse brain (axial orientation, 117 μm × 117 μm in plane resolution, 1 mm slice thickness, and 2 signal averages) acquired at 16.4 T. (**g**) *In vivo* ultrahigh-resolution mouse brain image (102 μm × 102 μm in plane resolution, 0.5 mm slice thickness and 4 signal averages) acquired from a different mouse at 9.4T for comparison.

The major goal of this study is to test the integrated TPM-MRI multimodal imaging system for simultaneously collecting TPM and MRI data inside the 16.4T magnet. To mimic the TPM imaging of animal brains, we placed a fixed brain of a CX3CR1 mouse at the center of the 16.4T MRI magnet. We used the long-distance remote TPM system to image the GFP expressing microglia cells. Even with the very low numerical aperture (NA = 0.26), the processes of the microglia and cell body are clearly visible (Fig. 3a). We went on to test if the operation of MRI would have any interference on the TPM imaging. In the test, we performed time lapsed TPM imaging of the same brain region. We turned on the MRI acquisition at frame 6 and turned it off at frame 16. To compare the TPM images and examine if there was any change or shift of the microglia cell images caused by the operation of MRI, we performed cross-correlation of the recorded TPM images. The results (Fig. 3b–d) show that there was no noticeable change in the TPM images and their position shift. This is expected as none of the in-MRI module was sensitive to the magnetic field, gradients and the pulsed radio frequency wave; and the actuators, detectors, and electronics were all housed outside the MRI magnet room shielded by the enclosure to reach near earth field strength (0.6–0.8 gauss measured inside the enclosure). We used the fine processes of the microglia cells to quantify the imaging performance. The cross-sectional plot (Fig. 3e) shows a FWHM of 1.47 ± 0.23 μm (n = 5), close to the theoretical value (1.35 μm) of a diffraction-limited focus, which again indicates the minimal aberration and distortion of the remote TPM-MRI system. Figure 3f demonstrates the ultrahigh resolution MRI (117 μm × 117 μm in-plane resolution, axial orientation) recorded at 16.4T from the same fixed mouse brain showing superior quality and detailed brain structures, and high similarity as the *in vivo* MRI (Fig. 3g) acquired in live mouse brain at 9.4T.

## Discussion and Conclusion

Although the combined optical imaging with MRI technique has been exploited and reported in the literature^56–58^, we present herein the first attempt to integrate multiphoton microscopic imaging with MRI at ultrahigh field. Overall, our preliminary results show that: 1) the long-distance remote TPM-MRI system can effectively protect the TPM components from the strong magnetic field; 2) the long-distance relay can enable near diffraction-limited imaging performance; 3) the novel TPM imaging system is fully compatible with the ultrahigh-field MRI system without interference from or to the MRI operation. These results confirm that the scheme of long-distance remote TPM imaging system is fully compatible and functional with ultrahigh-field MRI and will enable simultaneous TPM and MRI studies at 16.4T with the highest field horizontal animal MRI scanner currently in operation. Therefore, the TPM-MRI multimodal imaging system and design as presented herein should be applicable for a broad range of field strength covering all human and preclinical MRI scanners.

The limitation of this pilot study is that the employed objective lens is of low NA and there is no axial scanning capability. To improve the spatial resolution and the fluorescence emission collection efficiency, high NA water dipping low magnification objectives should be employed. Customized versions with all steel components replaced by MRI-compatible brass counterparts are commercially available.

The TPM imaging was limited to 2D imaging in this study. However, we can potentially combine the current system and three-dimensional volumetric imaging methods with slight modification^59^. For volumetric imaging, conventional systems typically employ piezo objective stage to translate the objective to image different depths. As piezo is incompatible with strong magnetic field, a better solution is to employ the remote focusing scheme^60^, which allows the wavefront control unit to be far from the objective lens (i.e., also housed inside the shielding enclosure outside the magnet room). Moreover, some of the remote focusing modules allow high-speed volumetric imaging^59^, which would be very useful for the simultaneous calcium imaging of neurons and astrocytes and morphology imaging of vessel dilation or contraction.

The TPM imaging only detects the optical signal from a small portion of cortical region of interest. In contrast, MRI could image the entire brain using a RF head volume coil. To study the cross correlation between the TMP and MRI measurements with high fidelity, an alternative approach is to employ a small RF surface coil^61^ located at the top of the brain region covered by the TPM imaging. This configuration can significantly reduce the MRI field of view (FOV) and maximize the MRI detection sensitivity, potentially push the isotropic fMRI resolution to 50–150 micron at 16.4T. Such a fMRI voxel size will be much smaller than the FOV of TPM imaging, resulting in a large number of fMRI voxels within the TPM imaging FOV for studying the spatial correlation between the measurements from two modalities at the level of single neuron or neuron population.

Another technical aspect is how to co-register the MRI and TPM images with different spatial resolution. One option is to collect ultrahigh resolution vasculature MRI^62^, then co-register it to the optical vasculature images based on the characterized vascular landmarks and multiple-dimensional geometric nonlinear transformation for the same brain.

In summary, this pilot study paved the way for simultaneous ultrahigh-field MRI and high-resolution TPM, which is anticipated to provide new insights about the electrophysiological basis of the fMRI BOLD signal and the functional mapping specificity for studying the dynamics of neural circuitry and functional connectivity across multiple scales in time and space, which is essential for understanding how brain functions. The same TPM-MRI multimodal imaging approach can be extended to perform *in vivo* TPM imaging with other types of MRI including anatomic structure and diffusion MRI as well as *in vivo* magnetic resonance spectroscopy imaging in the same brain. This imaging capability will provide new opportunities to study and understand the comprehensive relationships between brain structural and connectivity, neuronal activity and dynamics, brain function and cellular energy metabolism in supporting brain function.

## Materials and Methods

### Ultrahigh field animal MRI system

The *ex vivo* TPM-MRI experiments were conducted in a 16.4T horizontal animal magnet (Magnex Scientific, Abingdon, UK; 12 cm diameter of usable bore size) interfaced to a Varian INOVA console (Palo Alto, CA, USA) using a ^1^H RF surface coil (2 cm diameter) operated at 700 MHz for performing MRI. The *in vivo* mouse brain MRI was acquired using a 9.4T horizontal animal magnet (Magnex Scientific, Abingdon, UK; 15 cm diameter of usable bore size) interfaced to a Varian INOVA console (Palo Alto, CA, USA). The animal procedures and experiments were conducted in accordance with the National Research Council’s Guide for the Care and Use of Laboratory Animals and under the protocols approved by the Institutional Animal Care and Use Committee of University of Minnesota.

### Optical system

The laser system was a 80 MHz Ti:Sapphire Oscillator (MaiTai, Spectra-Physics, CA, USA). We employed a pair of 5 mm Galvo scanning mirrors (6215 H, Cambridge Technology, MA, USA) for raster scanning. Two pairs of relay lenses (f = 125 mm and f = 500 mm; f = 1900 mm and f = 1900 mm) imaged the Galvo mirrors onto the back focal plane of the objective lens. We used a simple iron shield to enclose the optical system and the key electronics (PMT, PMT signal amplifier, Galvo controller), which effectively reduced the residual magnetic field strength from 5–10 gauss to ≤0.8 gauss, very close to the earth’s magnetic field.

### *Ex vivo* brain sample

We used a fixed CX3CR1 mouse brain as the test sample. The mouse brain was placed inside a Petri dish, which was then filled with 2% agar. The fixed mouse brain was positioned in the focal area of the objective lens for the TPM-MRI measurement.
